# Supplementary material for: Phenomenological Changes Associated with Deep Brain Stimulation for Obsessive Compulsive Disorder: A Cognitive Appraisal Model of Recovery
Source: Brain Sci. 2023 Oct 10;13(10):1444. doi: 10.3390/brainsci13101444 (PMC10605199; doi:10.3390/brainsci13101444)
Supplement: Supplementary file 1 [file brainsci-13-01444-s001.zip › Supplementary/Supplementary 1_Participant Interview.pdf]

1. Can you tell me a bit about your life **before DBS treatment**?

2. Before we go into specifics, can you give me a brief overview of the **major changes** you have noticed in your life following DBS therapy?

In subsequent questions, when we discuss changes that have occurred, it would be useful if you can provide specific examples. You can go into as much detail as you like. I want you to consider changes that have affected you in a positive way, and changes that have affected you in a negative way. If there are changes that seem idiosyncratic or insignificant, these may indeed be important, and I would like to hear about all of the changes you have experienced. Throughout 2020, the COVID lockdown may have affected your thoughts, behaviours and actions in relation to the following questions; please think back and refer to times before COVID-19 lockdown.

Effects of DBS on the self

3. Have aspects of your **thoughts, mood or behaviour** changed?

4. Do you think there have been changes to **who you are** as a person, if so how do you evaluate these changes?

5. Have you experienced changes to how you **view and evaluate yourself**?

Effects of DBS on experience of the external word

6. Has your **perspective** of the world and the way in which you **engage** with, and **experience** the world around you changed?

Effects of DBS on the interpersonal world

7. Has the way in which you **interact** with others changed, if so, how has this affected your relationships?

Effect of DBS towards attitudes of wellbeing

8. What is your overall impression of DBS therapy and the associated changes to your **wellbeing** and **quality of life**?

9. Has anything changed in relation to how you **view your illness**?

Final comments

10. Please discuss any **recommendations** that may improve DBS therapy at any stage of the clinical care process for yourself or others

11. Do you have any **final comments** that are important to your experience of DBS treatment?

In subsequent questions, when we discuss changes that have occurred, it would be useful if you can provide specific examples. You can go into as much detail as you like. I want you to consider changes that have affected _____ in a positive way, and changes that have affected _____ in a negative way. If there are changes that seem idiosyncratic or insignificant, these may indeed be important, and I would like to hear about all of the changes you are aware of. Throughout 2020, the COVD lockdown may have affected _____ thoughts, behaviours and actions in relation to the following questions; please think back and refer to times before COVID-19 lockdown.

Background

1. What is your relationship to ____?

2. How many hours would you care for their needs
   i) Before DBS?
   ii) After DBS?

3. Did/ have you lived with____
   i) Before DBS?
   ii) After DBS?

4. What did/does your care consist of
   i) Before DBS?
   ii) After DBS?

Effects of DBS on the self

5. Have aspects of _____ **thoughts, mood or behaviour** changed?

6. Do you think there have been changes to **who** _____ **is** as a person, if so how do you evaluate these changes?

Effects of DBS on experience of the external word

7. Has _____ **perspective** of the world and how they **engages** with, and **experience** the world around them changed?

Effects of DBS on the interpersonal world

8. Has the way in which _____ **interacts** with others changed, if so, how has this affected their relationships, including with yourself?

Effect of DBS towards attitudes of wellbeing

9. What is your overall impression of DBS therapy and the associated changes to _____ **wellbeing** and **quality of life**?

Effect of DBS on carers

10. How have changes to _____ way of being following DBS affected **your life**?

Final comments

11. Please discuss any **recommendations** that may improve DBS therapy at any stage of the clinical care process for DBS patients or carers

12. Do you have any **final comments** that are important _____ and your experience of DBS treatment?

COREQ item	Description
**1. Interviewer**	N.A.
**2. Credentials**	BSc (Hon)
**3. Occupation**	PhD student, Clinical Trial Coordinator
**4. Gender**	Female
**5. Experience and training**	5 years working in the field of DBS for movement and psychiatric conditions, mentoring and training conducted by S.R. on interview and analysis techniques.
**6. Relationship established**	Discussions via phone- explanatory sessions
**7. Participant knowledge of the interviewer**	Participants were aware the interviewer was completing the investigations for a PhD project and working on the clinical trial. Participants were informed that data collection and analysis was independent to their clinical care, and only de-identified data was to be shared with their clinical team.
**8. Interviewer characteristics**	No bias was identified or reported
**9. Methodological orientation and theory**	Thematic, content, and interpretive phenomenological analysis (IPA)
**10. Sampling**	Purposeful sampling
**11. Method of approach**	Invited to participate by psychiatrist, then followed up via phone by the interviewer.
**12. Sample size**	14
**13. Non-participation**	0
**14. Setting of data collection**	University
**15. Presence of non-participants**	No
**16. Description of sample**	Six people with OCD receiving DBS therapy who had reached clinical response (≥35 % improvement in symptoms), and six close family members of the people with OCD who had been involved in their care prior to DBS and post DBS.
**17. Interview guide**	Interview guide provided in Supplementary Material 1. All participants were asked the same core questions, prompts and follow up questions varied.
**18. Repeat interviews**	No
**19. Audio recording**	Interviews were audio recorded
**20. Field notes**	Notes regarding general impressions were taken
**21. Duration**	1 hour
**22. Data saturation**	Yes
**23. Transcript returned**	Participants reviewed codes generated from the interview transcript
**24. Number of data coders**	Two (N.A., S.R.)
**25. Description of the coding tree**	A set of codes was developed for each transcript, through an inductive and latent approach, the transcript and set of codes were reviewed several times and refined as necessary in an iterative manner. Each code was defined, participants reviewed the set of codes and definitions, and made any necessary changes.
**26. Derivation of themes**	The set of codes for each transcript were collectively reviewed to develop a thematic map with themes and sub-themes. Each theme was defined considering experiences from each individual and thus each set of codes relating to that theme. Themes were expanded using sub-themes, each set of themes was reviewed for internal homogeneity and external heterogeneity, however interdependencies between different themes and sub-themes were present.
**27. Software**	Qualtrics

**28. Participant checking**	Participants checked codes, minor changes were made based on feedback, and participants confirmed agreement with codes.
**29. Quotations presented**	Yes
**30. Data and findings consistent**	The theoretical framework and model proposed show global patterns in the data, there was an exception to these changes. One participant (P6) was unable to articulate meaningful changes during the interview, it is suspected that a comorbid autism spectrum disorder interfered with the DBS induced effects, ability to engage in further therapy to consolidate DBS induced effects, and the ability to express changes experienced. Another participant (P2) did not identify the multitude of changes others expressed. Thus, these participants represented a sub-group which deviated from the thematic map. Yet, these exceptions are discussed and represented for transparency.
**31. Clarity of major themes**	Three groups of themes are presented, themes and sub-themes were categorised into 1) psychopathological changes, 2) the self and identity and 3) changes identified by carers.
**32. Clarity of minor themes**	Sub-themes are presented, with a description, any cases which were not in agreement with the themes/ sub-themes are reported.
