# Supplementary material for: Phenomenological Changes Associated with Deep Brain Stimulation for Obsessive Compulsive Disorder: A Cognitive Appraisal Model of Recovery
Source: Brain Sci. 2023 Oct 10;13(10):1444. doi: 10.3390/brainsci13101444 (PMC10605199; doi:10.3390/brainsci13101444)
Supplement: Supplementary file 1 [file brainsci-13-01444-s001.zip › Supplementary/Supplementary 2_Carer interview.pdf]

In subsequent questions, when we discuss changes that have occurred, it would be useful if you can provide specific examples. You can go into as much detail as you like. I want you to consider changes that have affected _____ in a positive way, and changes that have affected _____ in a negative way. If there are changes that seem idiosyncratic or insignificant, these may indeed be important, and I would like to hear about all of the changes you are aware of. Throughout 2020, the COVD lockdown may have affected _____ thoughts, behaviours and actions in relation to the following questions; please think back and refer to times before COVID-19 lockdown.

Background

1. What is your relationship to ____?

2. How many hours would you care for their needs
   i) Before DBS?
   ii) After DBS?

3. Did/ have you lived with____
   i) Before DBS?
   ii) After DBS?

4. What did/does your care consist of
   i) Before DBS?
   ii) After DBS?

Effects of DBS on the self

5. Have aspects of _____ **thoughts, mood or behaviour** changed?

6. Do you think there have been changes to **who** _____ **is** as a person, if so how do you evaluate these changes?

Effects of DBS on experience of the external word

7. Has _____ **perspective** of the world and how they **engages** with, and **experience** the world around them changed?

Effects of DBS on the interpersonal world

8. Has the way in which _____ **interacts** with others changed, if so, how has this affected their relationships, including with yourself?

Effect of DBS towards attitudes of wellbeing

9. What is your overall impression of DBS therapy and the associated changes to _____ **wellbeing** and **quality of life**?

Effect of DBS on carers

10. How have changes to _____ way of being following DBS affected **your life**?

Final comments

11. Please discuss any **recommendations** that may improve DBS therapy at any stage of the clinical care process for DBS patients or carers

12. Do you have any **final comments** that are important _____ and your experience of DBS treatment?
