# Supplementary material for: Phenomenological Changes Associated with Deep Brain Stimulation for Obsessive Compulsive Disorder: A Cognitive Appraisal Model of Recovery
Source: Brain Sci. 2023 Oct 10;13(10):1444. doi: 10.3390/brainsci13101444 (PMC10605199; doi:10.3390/brainsci13101444)
Supplement: Supplementary file 1 [file brainsci-13-01444-s001.zip › Supplementary/Supplementary 3_COREQ table.pdf]

COREQ item	Description
**1. Interviewer**	N.A.
**2. Credentials**	BSc (Hon)
**3. Occupation**	PhD student, Clinical Trial Coordinator
**4. Gender**	Female
**5. Experience and training**	5 years working in the field of DBS for movement and psychiatric conditions, mentoring and training conducted by S.R. on interview and analysis techniques.
**6. Relationship established**	Discussions via phone- explanatory sessions
**7. Participant knowledge of the interviewer**	Participants were aware the interviewer was completing the investigations for a PhD project and working on the clinical trial. Participants were informed that data collection and analysis was independent to their clinical care, and only de-identified data was to be shared with their clinical team.
**8. Interviewer characteristics**	No bias was identified or reported
**9. Methodological orientation and theory**	Thematic, content, and interpretive phenomenological analysis (IPA)
**10. Sampling**	Purposeful sampling
**11. Method of approach**	Invited to participate by psychiatrist, then followed up via phone by the interviewer.
**12. Sample size**	14
**13. Non-participation**	0
**14. Setting of data collection**	University
**15. Presence of non-participants**	No
**16. Description of sample**	Six people with OCD receiving DBS therapy who had reached clinical response (≥35 % improvement in symptoms), and six close family members of the people with OCD who had been involved in their care prior to DBS and post DBS.
**17. Interview guide**	Interview guide provided in Supplementary Material 1. All participants were asked the same core questions, prompts and follow up questions varied.
**18. Repeat interviews**	No
**19. Audio recording**	Interviews were audio recorded
**20. Field notes**	Notes regarding general impressions were taken
**21. Duration**	1 hour
**22. Data saturation**	Yes
**23. Transcript returned**	Participants reviewed codes generated from the interview transcript
**24. Number of data coders**	Two (N.A., S.R.)
**25. Description of the coding tree**	A set of codes was developed for each transcript, through an inductive and latent approach, the transcript and set of codes were reviewed several times and refined as necessary in an iterative manner. Each code was defined, participants reviewed the set of codes and definitions, and made any necessary changes.
**26. Derivation of themes**	The set of codes for each transcript were collectively reviewed to develop a thematic map with themes and sub-themes. Each theme was defined considering experiences from each individual and thus each set of codes relating to that theme. Themes were expanded using sub-themes, each set of themes was reviewed for internal homogeneity and external heterogeneity, however interdependencies between different themes and sub-themes were present.
**27. Software**	Qualtrics

**28. Participant checking**	Participants checked codes, minor changes were made based on feedback, and participants confirmed agreement with codes.
**29. Quotations presented**	Yes
**30. Data and findings consistent**	The theoretical framework and model proposed show global patterns in the data, there was an exception to these changes. One participant (P6) was unable to articulate meaningful changes during the interview, it is suspected that a comorbid autism spectrum disorder interfered with the DBS induced effects, ability to engage in further therapy to consolidate DBS induced effects, and the ability to express changes experienced. Another participant (P2) did not identify the multitude of changes others expressed. Thus, these participants represented a sub-group which deviated from the thematic map. Yet, these exceptions are discussed and represented for transparency.
**31. Clarity of major themes**	Three groups of themes are presented, themes and sub-themes were categorised into 1) psychopathological changes, 2) the self and identity and 3) changes identified by carers.
**32. Clarity of minor themes**	Sub-themes are presented, with a description, any cases which were not in agreement with the themes/ sub-themes are reported.
