# Supplementary figures and images for: Phenomenological Changes Associated with Deep Brain Stimulation for Obsessive Compulsive Disorder: A Cognitive Appraisal Model of Recovery
Source: Brain Sci. 2023 Oct 10;13(10):1444. doi: 10.3390/brainsci13101444 (PMC10605199; doi:10.3390/brainsci13101444)

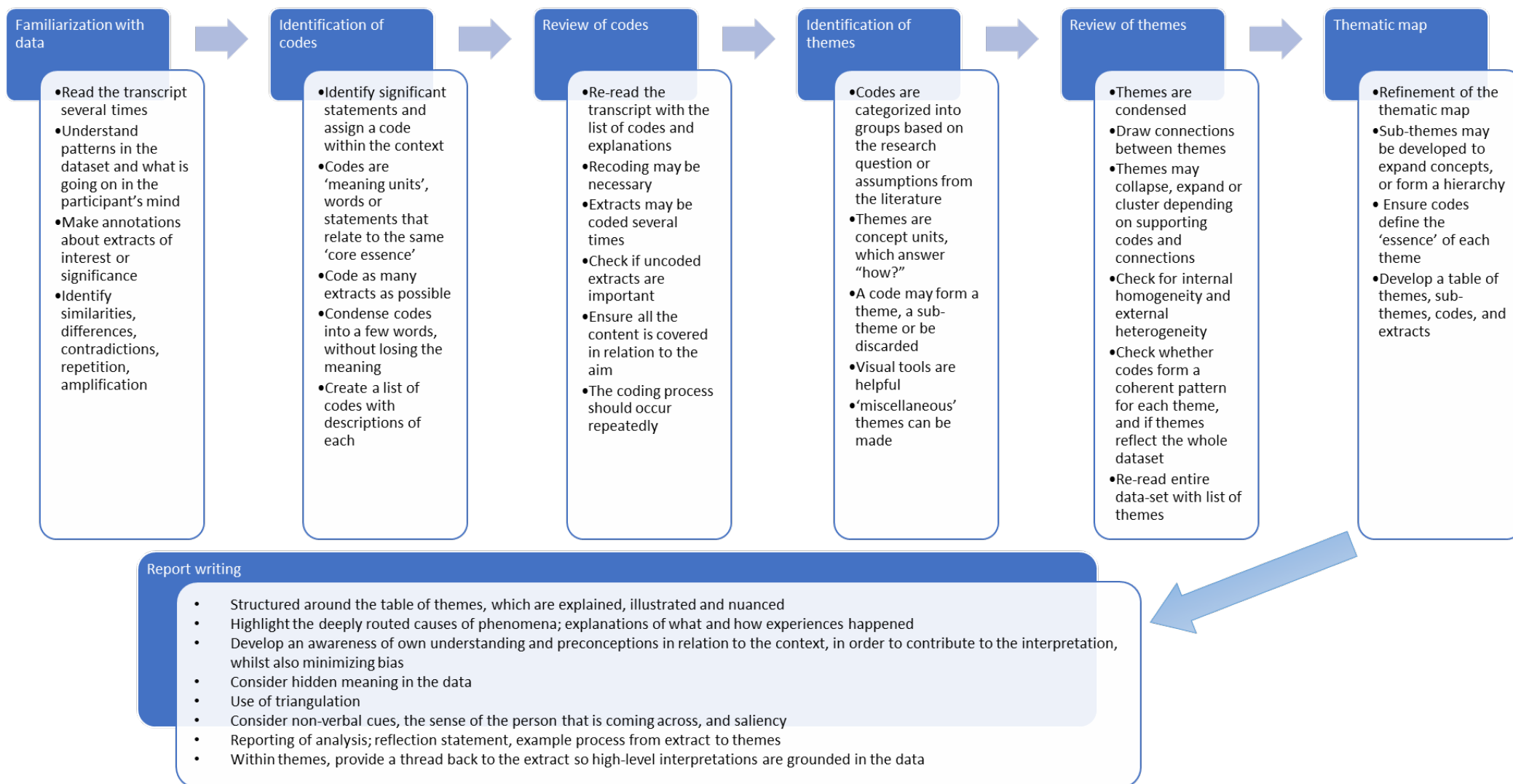

Supplement: Supplementary file 1 [file brainsci-13-01444-s001.zip › Supplementary/Supplementary 4_Thematic map stages.pdf]
